# Implementing VIRADS score for image-guided assessment of muscle invasiveness in bladder cancer pre-TURBT: An updated meta-analysis

**DOI:** 10.1080/20905998.2024.2442256

**Published:** 2024-12-24

**Authors:** Ahmad R. Al-Qudimat, Doaa Sabir, Muna Elamin, Mica Ching, Seif B. Altahtamouni, Kalpana Singh, Ibrahim A. Khalil, Khalid Alrumaihi

**Affiliations:** a Surgical Research Section, Department of Surgery, Hamad Medical Corporation, Doha, Qatar; b Public Health college, Health Science, Qatar University, Doha, Qatar; c Department of Urology, Hamad Medical Corporation, Doha, Qatar; d Department of Urology, College of Medicine, Qatar University, Doha, Qatar

**Keywords:** Bladder cancer, urothelial carcinoma, VI-RADS, systematic review, meta-analysis

## Abstract

**Background:**

Bladder urothelial carcinoma is the most prevalent malignancy of the urinary system worldwide. Accurate staging of bladder cancer, particularly distinguishing between non-muscle-invasive bladder cancer (NMIBC) and muscle-invasive bladder cancer (MIBC), is essential for determining appropriate treatment. This systematic review and meta-analysis aimed to evaluate the diagnostic accuracy, sensitivity, and specificity of the vesical imaging-reporting and data system (VI-RADS) scoring system using multiparametric MRI in differentiating NMIBC from MIBC.

**Methods:**

A systematic review and meta-analysis were conducted following PRISMA guidelines. Comprehensive searches were performed in PubMed, Web of Science, Embase, and Cochrane databases up to December 2023. Studies that evaluated the diagnostic accuracy of the VI-RADS scoring system using multiparametric magnetic resonance imaging (MRI) to distinguish between NMIBC and MIBC were included. Data from eligible studies were extracted to calculate pooled sensitivity and specificity, and heterogeneity was assessed using meta-regression and subgroup analyses by using STATA V17.0.

**Results:**

A total of 31 studies, comprising 3,798 bladder cancer patients, were included in the meta-analysis. The pooled sensitivity and specificity for predicting MIBC using a VI-RADS cutoff score of ≥ 3 was 89%, with moderate heterogeneity observed across studies. Subgroup analysis revealed variations in diagnostic performance based on geographic location (North America, Europe, and Asia), MRI technical parameters, and study design. Studies utilizing 3.0 Tesla MRI scanners and those involving multiple radiologists demonstrated higher diagnostic accuracy.

**Conclusion:**

The VI-RADS system demonstrates high diagnostic accuracy in distinguishing between NMIBC and MIBC, with a cutoff score of ≥ 3 yielding optimal sensitivity and specificity. Its integration into clinical practice has the potential to reduce the need for invasive procedures, improve staging accuracy, and expedite treatment decisions. Future research should focus on standardizing MRI protocols and further validating these findings across diverse clinical settings to enhance the utility of VI-RADS in bladder cancer management.

## Introduction

Bladder urothelial carcinoma, the most prevalent pathological type of bladder cancer, is the most common malignancy of the urinary system globally [1]. In the European Union, the age-adjusted incidence of bladder cancer is 20.0 per 100,000 in men and 4.6 per 100,000 in women [2]. Histologically, transitional cell carcinoma is the predominant type, classified into low and high-grade, with high-grade tumors further categorized as non-muscle-invasive (NMIBC) or muscle-invasive bladder cancer (MIBC) [3]. Approximately 75% of bladder cancer cases are diagnosed as NMIBC [2].

The standard treatment protocol for bladder tumors is primarily based on accurate staging, particularly distinguishing between NMIBC and MIBC. Currently, the differentiation is achieved through transurethral resection of the bladder tumor (TURBT) followed by histopathological examination [2]. However, the risk of under-staging post-TURBT ranges from 20% to 25%, depending on the surgeon’s experience [3], and muscle infiltration is missed in about 25% of invasive bladder cancer cases due to superficial resections not involving the muscularis propria layer [4]. Consequently, urologists often perform re-TURBT on patients with T1 tumors to confirm the diagnosis and avoid missing a higher-stage cancer [5]. Additionally, discrepancies can arise among radiologists regarding the extent of muscle invasiveness and among pathologists concerning the grade and stage of the disease [3].

TURBT carries significant risks, including bladder perforation, discomfort, and delays in radical treatment for those who need it urgently. Therefore, there is a pressing need for more accurate, rapid, and non-invasive staging techniques to enhance bladder cancer outcomes [6]. Preoperative prediction of muscle invasion through cross-sectional imaging could be a valuable alternative [7]. Although computed tomography (CT) of the abdomen and pelvis is the standard imaging method for staging MIBC and detecting extravesical extension (T3 or T4), its sensitivity for detecting intravesical tumors and quantifying muscle invasion (T1 or T2) is low [8]. Recently, multiparametric magnetic resonance imaging (mp-MRI) has been introduced into the diagnostic pathway of bladder cancer, leading to the development of the vesical imaging-reporting and data system (VI-RADS) score. The VI-RADS score significantly enhances preoperative diagnostic accuracy in distinguishing between NMIBC and MIBC by detecting the depth of tumor invasion, thereby improving the effectiveness of TURBT [9]. The VI-RADS scores range from 1 to 5 [1]: Muscle invasion is highly unlikely [2], Muscle invasion is unlikely [3], Muscle invasion is equivocal [4], Muscle invasion is likely, and [5] Muscle invasion beyond the bladder is very likely [10]. Implementing this scoring system could potentially reduce the number of patients undergoing TURBT and eliminate the need for a second resection in individuals with high-risk tumors resulting in lower complication rates, and shorter time to receive definitive treatment [2].

Despite the growing evidence supporting the use of VI-RADS, there is a significant gap in the literature. Previous systematic reviews have analyzed VI-RADS cut-off scores of ≥ 3 for staging bladder cancer using data from 20 studies [11]. However, this systematic review updates the existing body of research by including an additional 11 studies, expanding the total to 31. This systematic review and meta-analysis aimed to evaluate the diagnostic accuracy, sensitivity, and specificity of VI-RADS in differentiating NMIBC from MIBC using a VI-RADS cut-off score of 3.

## Methods

We performed the current systematic review and meta-analysis according to Preferred Reporting Items for Systematic Reviews and Meta-Analyses (PRISMA) guidelines [12].

### Search strategy

We systematically searched PubMed, Web of Science, Embase, and Cochrane (from their commencements to Dec 2023), Utilizing a search strategy devised following the Mesh terms of Vesical Imaging-Reporting and Data System (VI-RADS), the following keywords were employed: (‘Vesical Imaging-Reporting and Data System’ OR ‘VI-RADS’ OR ‘VIRADS’). We used EndNote X 9.0 to manage the retrieved studies, with excluded duplications. Additionally, review articles and guidelines underwent manual scrutiny to identify additional eligible articles not captured in the initial searches.

### Study eligibility & selection

Based on the Patient-Comparator-Outcome-Study (PICOS) design criteria, the research question was formulated as follows: 1) Involving patients with bladder cancer; 2) Assessing the VI-RADS index test based on mp-MRI against the reference standard or comparator derived from histopathological results obtained through TURB, re-TURBT, or cystectomy; 3) Data extracted from eligible studies were utilized to generate crosstabs representing true positive (TP), false positive (FP), false negative (FN), and true negative (TN) rates as the primary outcome measure; 4) Cohort studies, including original articles or conference abstracts, were considered for inclusion.

Exclusion criteria were applied to studies with alternative designs (e.g. review articles, case reports, editorials) or those lacking adequate information to calculate TP, FP, FN, and TN values for VI-RADS at a score cut-off ≥4.

As per the inclusion criteria, two authors (AA and SB) conducted thorough screening and evaluation of the relevant studies. In cases of differing opinions, discussions were held with the lead author (KA) until a mutual agreement was reached.

### Data extraction

Three authors (D.S, M.E, M.C.) independently extracted data using a standardized Excel sheet. The extracted information encompassed various study attributes, such as author names, publication years, study design, total study population, country of origin, and study duration. Additionally, the data included outcomes; the old standard for defining pathological MIBC involves several key technical and clinical considerations. These include the MRI acquisition settings, such as whether a 1.5 or 3 Tesla magnet is used, the thickness of the T2-weighted imaging (T2WI) slices, the specific b-values for diffusion-weighted imaging (DWI), and the temporal resolution for dynamic contrast-enhanced (DCE) MRI. Other important details include how the MRI scans are interpreted: the number of readers involved, the experience of the senior reader, the combined experience of all genitourinary MRI (GU-MRI) readers, and whether or not they were aware of the patient’s clinical history. The VI-RADS score cutoff for determining MIBC (score ≥3) is also critical in assessment.

### Quality assessment

In this meta-analysis, the Newcastle-Ottawa Scale (NOS) was used [13]. It is a widely used tool for assessing the quality of non-randomized studies. It evaluates studies based on three main categories: selection, comparability, and outcome (for cohort studies) or exposure (for case-control studies). The selection category assesses the representativeness and selection of cohorts, ascertainment of exposure, and the demonstration that the outcome of interest was not present at the start of the study. Comparability evaluates the study’s ability to control confounding factors. The outcome or exposure category examines the assessment of the outcome, the adequacy of follow-up, and whether the follow-up period was sufficient for the outcomes to occur. Each study can score up to a maximum of nine stars, with higher scores indicating higher quality and a lower risk of bias. The NOS helps in systematically comparing studies and identifying those with robust methodologies that provide reliable and generalizable results.

### Statistical analysis

We employed the Metadata function within Stata to consolidate test data regarding diagnostic accuracy. This function can support both bivariate random-effects and fixed-effects models, enabling the conduct of meta-regression analysis. The outcomes are presented through tables, forest plots, and/or summary receiver operating characteristic (SROC) plots [14]. In instances where covariates are absent, it computes unexplained heterogeneity, considering the between-study variance utilizing the I2 metric. The evaluation of publication bias was carried out through the utilization of Deeks’ funnel plot analysis. Our methodology incorporated Cochran’s Q test and Higgins I2 test [15], in addition to the inconsistency index, to identify heterogeneity in the pooled estimates [16]. A substantial level of heterogeneity was indicated when the I2 index surpassed 50%. To investigate the origins of inter-study heterogeneity, we undertook meta-regression and categorized the studies included into subgroups based on several parameters such as sample size (≥70 vs. <70), study design (prospective vs. retrospective), number of centers (single-center vs. multi-center), country (Australia, Europe, Asia, and North America), mean age (≥70 vs. <70), male percentage (≥80% vs. <80%), MIBC prevalence (≥35% vs. <35%), magnetic strength (3.0 Tesla, 1.5 Tesla, or mixed), number of individuals (Yes vs. No), and number of consensuses (≤2 readers vs. more than 2 readers). All statistical analyses were conducted using STATA 17.0, with a significance level of 0.05 set for subgroup analysis.

## Results

### Search result

**Figure 1.**
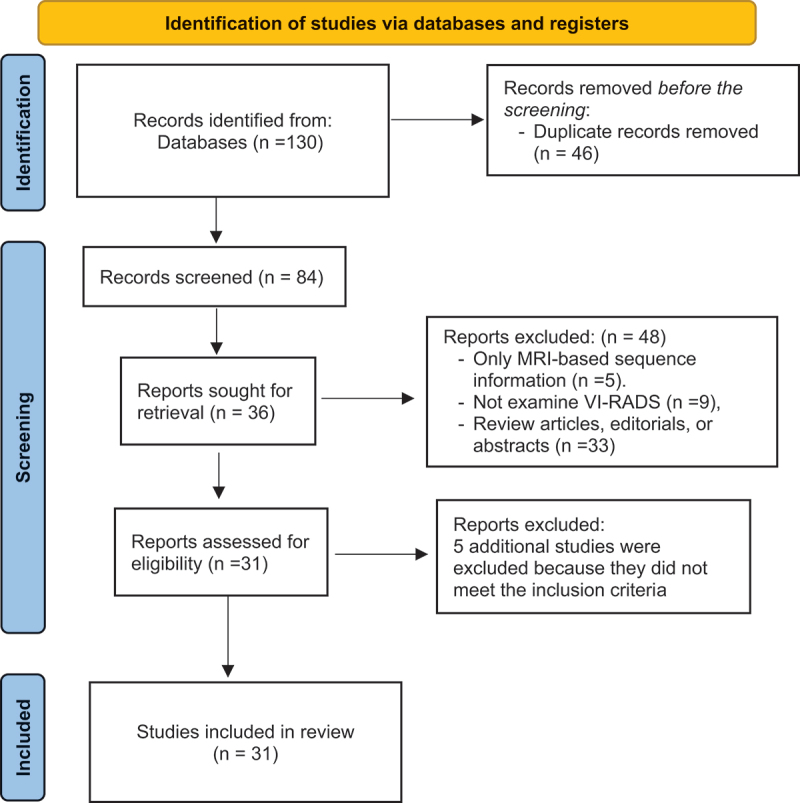
PRISMA diagram of literature search.

Initially, 130 records were identified across three databases: PubMed (97), Cochrane [7], and Embase [17]. After the removal of 46 duplicates, 84 unique records remained for screening. 53 studies were further excluded based on the evaluation of their titles or abstracts, or failure to meet the eligibility criteria. Following this, 31 full-text articles were assessed for eligibility. A total of 31 studies were originally included in the systematic review (Figure 1).

### Quality assessment

Out of the 31 studies, 15 studies are high-quality with a score of 9. Nine studies have moderate quality with a score of 6, typically retrospective in nature with limitations in controlling bias and confounding factors. 3 studies scored 7, suggesting reasonable quality but not as strong as the prospective high-quality studies. Overall, prospective studies scored higher and are considered more reliable due to better control over study conditions and follow-up, while retrospective studies generally presented moderate quality.

### Study characteristics

A total of 31 studies were included in our review, the meta-analysis comprised a total of 3798 patients diagnosed with bladder cancer. Among these, 2562 patients were male patients, while the remaining 1236 patients were female. 20 studies were retrospective, and 11 studies were prospective cohort studies. Table 1 provides an overview of all the details of the research characteristics. 1368 patients were diagnosed with MIBC. Most of the studies included in the study were Single center, while others present were multi-center.

**Table 1.** Characteristics of included studies.

Studies	Study design	Sample size N	Age mean±SD	Male N (%)	MIBC N (%)	No. of lesions	References	NOS
1. Barchetti G et al. [6]	Retrospective	75	69.6 ± 11.8	62(82.6)	22 (29.3)	103	TURBT, re-TURBT, radical cystectomy	[1–3]6
2. Kim SH et al. [17]	Retrospective	297	65.5 ± 12.8	222 (74.7)	132 (44.4)	339	TURBT, re-TURBT, partial/radical cystectomy	[1–3]6
3.Makboul M et al. [18]	Prospective	50	57.1 ± 7.3	46 (92)	18 [19]	50	TURBT, re-TURBT	[2–4]9
4. Ueno Y et al. [20]	Retrospective	74	72.8 ± 10.1	59 (87)	37 (50)	74	TURBT, re-TURBT	[1–3]6
5. Wang H et al. [21]	Retrospective	340	63.9 ± 10.2	296 (87)	85 [22]	340	TURBT, partial/radical cystectomy	[1–3]6
6. Ahn H et al. [7]	Retrospective	82	68.6 ± 9.8	73 (89)	19 (23.1)	82	TURBT, re-TURBT, radical cystectomy	[1–3]6
7. Arita Y et al. [23]	Retrospective	66	67.7 ± 9.8	60 (90.9)	17 (25.7)	66	TURBT, re-TURBT, radical cystectomy	[1–3]6
8. Del Giudice F et al. [24]	Prospective	231	Ns	Ns	62 (26.8)	231	TURBT	[2–4]9
9. Hong SB et al. [25]	Retrospective	32	70 ± 6.6	27 (84.3)	10 (31.2)	66	TURBT, re-TURBT, radical cystectomy	[1–3]6
10. Liu S et al. [26]	Retrospective	126	68 ± 11.6	104 (82.5)	50 (39.6)	126	TURBT, radical cystectomy	[1–3]6
11.Marchioni M et al. [27]	Retrospective	38	73.3 ± 10.7	27 (71)	7 (18.4)	68	TURBT, re-TURBT	[1–3]6
12. Sakamoto K et al. [22]	Retrospective	176	67.7 ± 10.8	125 (71)	46 (26.1)	176	TURBT, re-TURBT	[2,3]7
13. Ueno Y et al. [28]	Retrospective	91	73.2 ± 10.2	72 (79.1)	47 (51.6)	91	TURBT, radical cystectomy	[1–3]6
14. Vaz A et al. 2020 [8]	Retrospective	30	68 ± 8.8	19 (63.3)	8 (26.6)	30	TURBT	[1–3]6
15. Wang Z et al. [29]	Retrospective	220	65.6 ± 9.6	194 (88.1)	113 (51.3)	220	TURBT, partial/radical cystectomy	[2–4]9
16. Pizzi AD et al. [30]	Prospective	38	73.3 ± 10.7	27 (71)	7 (18.4)	38	TURBT, re-TURBT, radical cystectomy	[2–4]9
17. Akcay A et al. [4]	Prospective	73	67.7 ± 10.9	Ns	31 (42.4)	73	TURBT, radical cystectomy	[2,3]7
18. Metwally MI et al. [31]	Retrospective	331	61.9 ± 9.7	274 (82.7)	189 (57)	331	TURBT, re-TURBT and only TURBT*	[1–3]6
19. Li et al. [32]	Retrospective	80	60.71 ± 10.7	66 (82.5)	27 [31]	80	TURBT, cystectomy	[2–4]9
20.Ghanshyam et al. [3]	Prospective	86	Ns	67 (77.9)	34 (39.5)	Ns	TURBT, re-TURBT, radical cystectomy	[2,3]7
21.Del Giudice F et al. [19]	Retrospective	149	70 (62–75) *	118 (79)	109 (73.1)	149	TURBT, re-TURBT, radical cystectomy	[1–3]6
22.Erkoc et al. 2021 [33]	Retrospective	330	Ns	Ns	94 (28.5)	Ns	TURBT	[1–3]6
23.Gmeiner et al. [34]	Retrospective	59	68.2 ± 13.6	48 (81.3)	14 (27.5)	Ns	TURBT, re-TURBT, radical cystectomy	[1–3]6
24. Huang et al. [35]	Retrospective	43	71	37(86)	20 (46.5)	Ns	TURBT, partial/radical cystectomy	[2–4]9
25.ElKaramany et al. [9]	Prospective	80	61.9 ± 12.1	64 (80)	24 [25]	Ns	TURBT, re-TURBT	[2–4]9
26. Oğuz et al. [5]	Prospective	70	70.7 ± 10.7	58 (82.8)	16 (22.8)	Ns	TURBT, re-TURBT	[2–4]9
27.Rysankova et al. [2]	Prospective	64	68 (62–75) *	41 (66)	Ns	72	TURBT, re-TURBT	[2–4]9
28.Bicchetti et al. [36]	Prospective	139	70 (64–76.5) *	103 (74.1)	42 (30.2)	139	TURBT, re-TURBT, radical cystectomy	[2–4]9
29. Kim et al. [37]	Prospective	159	70 (63–76) *	132 (83)	48 (30.2)	Ns	Radical cystectomy	[2–4]9
30. Cao et al. [1]	Prospective	73	68 ± 10	57 (78)	20 [18]	Ns	TURBT, re-TURBT, partial/radical cystectomy	[2–4]9
31. Kazan O et al. [38]	Retrospective	96	67.4 ± 11.7	84 (87.5)	20 (20.8)	Ns	TURBT, re-TURBT, radical cystectomy	[1–3]6

SD; Standard deviation, NOS; Newcastle-Ottawa Scale, * median and interquartile range (IQR), Ns; not stated.

### Technical & radiologist characteristics

Overview of the technical and radiologist characteristics across 31 studies, focusing on their VI-RADS values. The field strength of the MRI machines ranges from 1.5 to 3 Tesla, with T2-weighted imaging (T2WI) slice thickness varying between 2 to 6 mm. DWI b values differ significantly, spanning from 0 to 10,000 s/mm^2^. The temporal resolution for DCE-MRI also varies, from every 5 seconds to multiple acquisitions over several minutes. The number of radiologists involved per study ranges from 1 to 7, with their years of experience spanning from 5 to over 32 years. Reporting methodologies vary, with some studies using individual reporting and others employing agreement reporting. Additionally, the studies demonstrate diverse approaches to reader blindness. VI-RADS values, indicating the radiologist’s assessments, predominantly range between 2 and 5 (Table 2).

**Table 2.** Technical & radiologist characteristics.

Study No.	*Technical characteristics*				*Radiologist characteristics*					*VI-RADS values*
	Field strength (Tesla)	T2WI slice thickness (mm)	DWI b values (s/mm2)	DCE MRI temporal resolution (s)	No. of readers	Experience – years	Individual reporting	Agreement reporting	blindness	
*1*	3	3 to 4	0, 88, 1000, 2000	Every 5 s	2	10,5	Yes	No	Yes	1 to 5
*2*	3	3	0, 10000	Every 30 s for 4–6 acquisitions	2	12,6	No	Yes	Yes	3,4
*3*	1.5	3	0, 400, 800, 10000	At 20, 70, 180 s	2	Ns	No	Yes	Ns	3
*4*	1.5, 3	4	0, 1000	At 40, 80, 120, 160, 200 s	5	Ns	Yes	Yes	Yes	3,4
*5*	3	4	0, 1000	5 acquisitions between 20 and 131 s	2	32,8	No	Yes	Yes	3
*6*	3	4	0, 1000	Ns	2	21,5	No	Yes	Yes	3
*7*	1.5	2	0, 1000	Every 40 s	2	18,12	Yes	No	Yes	3,4
*8*	3	3 to 4	0, 88, 1000, 2000	Every 5 s	2	25,12	No	Yes	Yes	3
*9*	3	4	0, 50, 800, 1000	6 acquisitions every 30 s	3	24,16,7	No	Yes	Yes	2,3
*10*	3	4,5,6	800, 1000	5–6 acquisitions between 20–131 s	2	Ns	No	Yes	Yes	2 to 5
*11*	3	3 to 4	0, 600, 1000, 1500, 2000	Every 31.2 s for 3.2 min	3	Ns	No	Yes	Yes	4
*12*	3	3.5, 4 to 5	0, 1000; 0, 2000	Ns	2	15,9	No	Yes	Yes	3,4
*13*	1.5, 3	4	0, 1000	At 30,60, 90, 120, 180 s	7	Ns	Yes	No	Yes	3,4
*14*	1.5	Ns	50, 400, 800	At 60, 300 s	2	13	No	Yes	Yes	3,4
*15*	3	Ns	Ns	Ns	2	Ns	No	Yes	Yes	3,4
*16*	3	4	0, 600, 1000, 1500, 2000	Ns	2	10,15	Yes	No	Ns	4
*17*	1.5	3	0, 1800	Every 8 s	2	20,5	Yes	No	Yes	3,4
*18*	1.5	4,5,6	0, 800, 1000	Every 5 s	4	10,12,13,16	Yes	Yes	Yes	3,4
*19*	3	4	0, 800	Ns	2	16,8	No	Yes	Yes	3
*20*	3	Ns	0, 88, 1000, 2000	Every 5 s	1	Ns	No	Yes	Ns	3,4
*21*	3	Ns	0, 800, 1000, 2000	Every 5 s	2	>10,>10	No	Yes	Yes	5
*22*	1.5	Ns	Ns	Ns	2	>5,>5	No	Yes	Ns	3
*23*	3	3	50, 400, 800	Every 6.22 s	2	>10	Yes	Yes	Yes	3
*24*	3	3	Ns	Ns	2	15,3	Yes	Yes	Yes	3,4
*25*	1.5	3	0, 400,800, 1,000	5 acquisitions between 30 and 1901 s	Ns	Ns	Ns	Ns	Ns	2,3
*26*	1.5	4, 1 to 2	50, 800, 1400	At 30, 90, 180 s	2	9,10	No	Yes	Yes	4
*27*	1.5	3	0, 500, 1000	6 acquisitions every 25 s	1	20	No	No	Ns	3,4
*28*	3	3 to 4	50, 500, 800, 1000	Every 5 to 9 s	2	>9, <6	Yes	Yes	Yes	3,4
*29*	3	Ns	Ns	Ns	2	Ns	No	Yes	Yes	3,4,5
*30*	3	3	0,200,800,1500,2000	3 min 52 s/7. 7 s	5	17,15,9,7,3	Yes	Yes	Yes	3,4
*31*	1.5	3,4,3	50, 800, 1000	6 ms	2	14,10	Yes	Yes	Yes	3,5

Ns; not stated.

**Figure 2.**
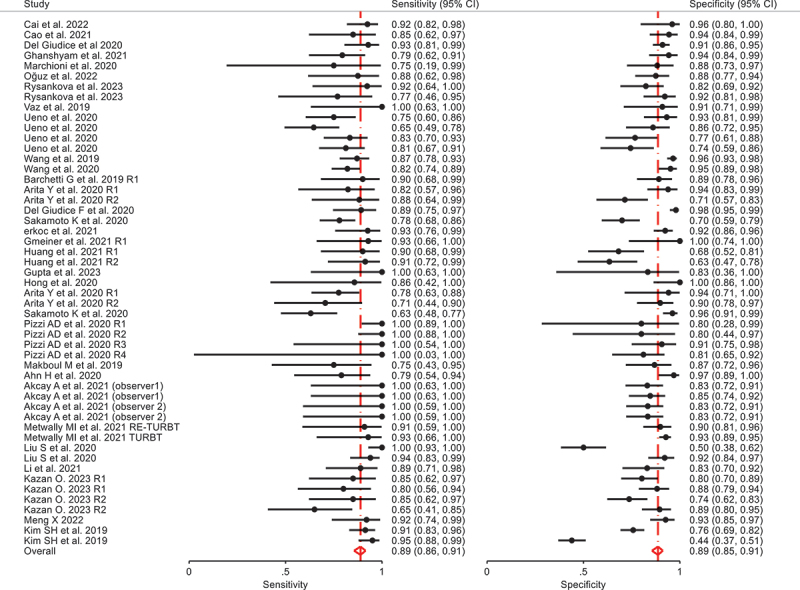
Forest plot of diagnostic accuracy of studies using VI-RADS predicting MIBC.

**Figure 3.**
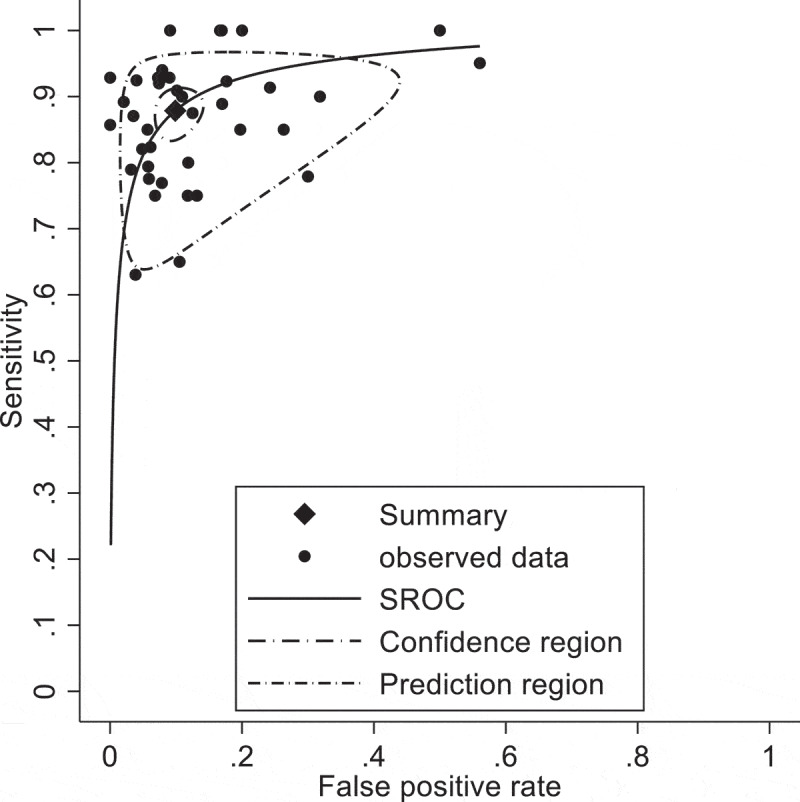
HSROC for diagnostic performance of studies using VI-RADS predicting MIBC.

All the studies enrolled reported diagnostic accuracy information for VI-RADS cut-of ≥3 for MIBC. Pooled paired Sensitivity and Specificity were 0.89 (95% CI 0.86–0.91) and 0.89 (95% CI 0.85–0.91) respectively (Figures 2, 3). There is a moderate level of heterogeneity in terms of specificity (σ2 = 0.55, I2 = 54.1%) and sensitivity σ2 = 0.36, I2 = 22.7%). We have more heterogeneity in terms of specificity as compared to sensitivity. The p-value of the LR test comparing the fitted random-effects to a fixed-effects model is < 0.0001. This indicates that the random effects are a better fit for the data.

Studies utilizing 3 Tesla MRI scanners demonstrated higher diagnostic accuracy compared to those using 1.5 Tesla MRI scanners. Pooled paired Sensitivity and Specificity for studies using 3 Tesla were 0.88 (95% CI 0.84–0.91) and 0.92 (95% CI 0.86–0.95) respectively (Table 3).

**Table 3.** Subgroups analysis of studies using VI-RADS predicting MIBC.

	Subgroups	Study (n)	Pooled sensitivity% (95% CI)	Pooled specificity% (95% CI)	Generalized I^2^
	Overall	25	0.89(0.86,0.91)	0.89(0.85,0.91)	36.2%
Sample size	<80	13	92(88 to 95)	89(82 to 94)	0.00%
	≥80	10	89(82 to 94)	86(76 to 96)	62.4%
Study design	Prospective	8	94(87 to 97)	88(72 to 95)	22.2%
	Retrospective	15	88(83 to 92)	88(82 to 92)	50.4%
No of centers	Single	19	91(86 to 94)	87(80 to 92)	45.6%
	Multi-centric	3	87(72 to 95)	92 (89 to 94)	0%
Age (Mean)	<70 years	14	88(81 to 93)	90 (84 to 94)	43.2%
	≥70 years	5	95(80 to 99)	85(70 to 93)	41%
Male percentage	<80%	9	87(80 to 90)	93(87 to 96)	45.8%
	≥80%	13	92(88 to 95)	83(73 to 90)	1%
MIBC	<35	14	92(87 to 95)	88(79 to 93)	49.6%
	≥35	9	91(83to 96)	88(78 to 94)	0.04%
Magnetic strength	1.5 Tesla	10	83(74to90)	88(85 to 91)	15.1%
	3 Tesla	15	88(84 to 91)	92(86 to 95)	35.9%
No. of consensuses*	≤2	20	88(84 to 91)	90(85 to 93)	38.9%
	>2	5	79(63 to 89)	91(86 to 95)	0.01%
Individual reporting^$^	No	15	87(83 to 90)	92 (87 to 95)	31.6%
	Yes	10	88(79 to 94)	87(81 to 91)	38.1%
Country	Australia	1	0.91(0.72, 0.99)	0.63(0.47, 0.78)	0%
	North America	1	0.79(0.54, 0.94)	0.97(0.89,1.00)	0%
	Europe	6	0.93(0.88,0.96)	0.91(0.88,0.94)	0%
	Asia	17	0.90(0.84,0.94)	0.87(0.78, 0.93)	50.7%

*Number of consensuses by reporting radiologists.

^$^Individual reporting radiologist.

### Consensuses and number of reporting radiologists

We found that the pooled sensitivity of VI-RADS when there is consensus between two or more radiologist is 0.88 (95% CI 0.84–0.91), with a pooled Specificity of 0.91 (95% CI 0.86–0.95), Nevertheless the individual reporting has a Pooled paired Sensitivity and Specificity of 0.88 (95% CI 0.79–0.94) and 0.87 (95% CI 0.81–0.91) respectively (Table 3).

### Gender distribution

**Figure 4.**
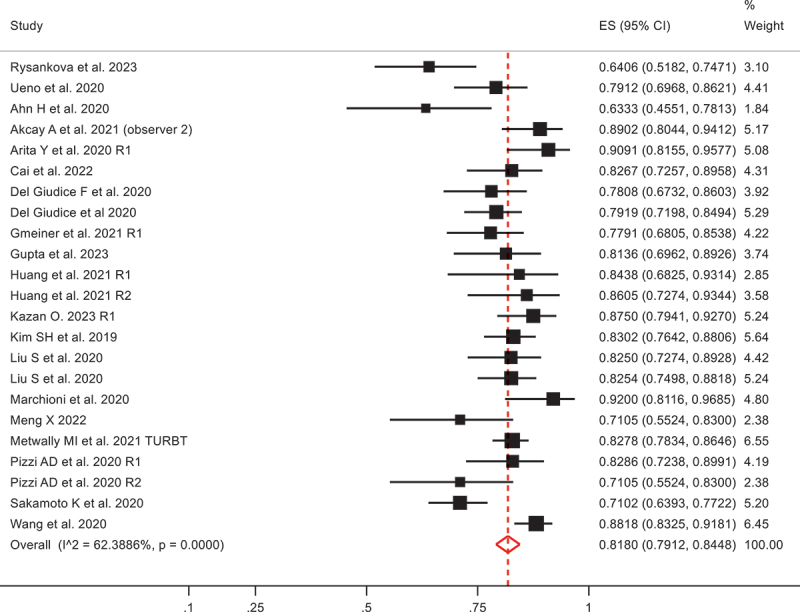
Forest plot of proportion rate of males.

Figure 4 depicts the specific proportions of males within each study, accompanied by their respective 95% exact confidence intervals. The overall prevalence of males under random-effects pooling was calculated to be 81.8% (95% CI: 79.1% to 84.5%), revealing a moderate level of significant heterogeneity (I^2^ = 62.4%, *p* < 0.0001) as demonstrated in Figure 2. Additionally, the prevalence range ranged from a minimum of 63.3% (95% CI: 45.5%-78.1%) to a maximum of 92% (95% CI: 81.2% to 96.9%).

### Geographic location

**Figure 5.**
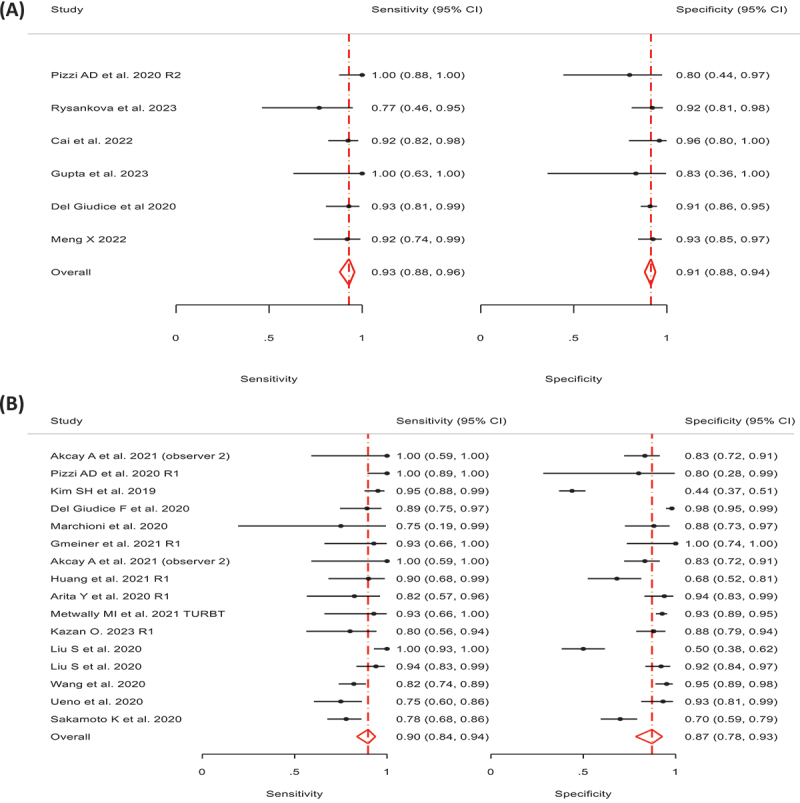
(a) Forest plot of diagnostic accuracy of studies using VI-RADS predicting MIBC for European countries (b) forest plot of diagnostic accuracy of studies using VI-RADS predicting MIBC for Asian countries.

A subgroup analysis was performed to investigate the impact of geographic location, revealing that North America (Table 3) exhibited lower sensitivity compared to regions such as Europe, Asia, and Australia. We also examined subgroup analyses based on sample size, age, gender, percentage of MIBC, study type, number of centers, magnetic strength, number of readers, and individual reporting (Figure 5).

### Publication bias

**Figure 6.**
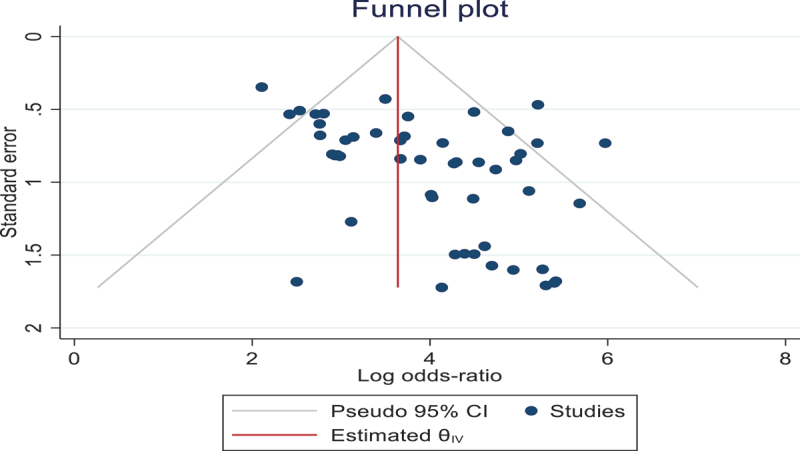
Deeks’ funnel plot for studies using VI-RADS predicting MIBC.

Deeks funnel plot as a means of detecting publication bias. The funnel plot displayed asymmetry, indicating publication bias within our study. However, recent studies have increasingly refrained from evaluating publication bias, acknowledging challenges in assessing reporting and publication biases in diagnostic accuracy studies (Figure 6).

## Discussion

This meta-analysis highlights the diagnostic utility of the Vesical Imaging-Reporting and Data System (VI-RADS) in detecting MIBC with a cutoff score of ≥ 3, showing excellent pooled sensitivity and specificity (89% and 84%, respectively). These findings underscore the high diagnostic accuracy of VI-RADS, consistent with earlier studies that established its clinical relevance [11,39].

For technical parameters and diagnostic accuracy, our analysis found that technical parameters, such as MRI magnetic field strength and T2-weighted imaging (T2WI) slice thickness, had a significant impact on the diagnostic accuracy of VI-RADS. Studies utilizing 3.0 Tesla MRI scanners with thinner T2WI slices (2–3 mm) outperformed those using 1.5 Tesla MRI scanners with thicker slices (4–5 mm) [11,39]. These findings align with those of Del Giudice et al., who reported that using higher-resolution MRI sequences improved sensitivity and specificity across studies [11,39]. mp-MRI played a crucial role in improving diagnostic precision. mpMRI, through its superior contrast resolution in soft tissues, allows for better differentiation between NMIBC and MIBC, as demonstrated in several studies included in our analysis [11,39]. This is consistent with reports by Wang et al. and Ueno et al., who noted the added value of combining T2WI, DWI, and DCE sequences for accurate bladder cancer staging [21,28,39].

Regarding reader variability and consensus reporting, this review revealed that diagnostic accuracy was higher when multiple radiologists performed consensus readings. Studies involving agreement among two or more radiologists demonstrated better sensitivity and specificity than those with single reader assessments [11,39](SR 1). For example, Meng et al. found that consensus readings by more than two radiologists improved diagnostic consistency, reducing inter-observer variability [39,40]. Del Giudice et al. also emphasized that collaborative assessments significantly improve diagnostic performance [11].

The role of reader experience was highlighted by several studies, including Akcay et al., who showed that diagnostic accuracy increased with the expertise of genitourinary (GU) radiologists [4,39]. Studies that involved radiologists with more than five years of GU MRI experience demonstrated higher diagnostic performance, emphasizing the importance of specialized training in applying VI-RADS [39]). Similar results were observed in Wang et al. (2020), where experienced readers provided more accurate MIBC detection, supporting the need for training programs to standardize the use of VI-RADS [21,39].

On the other hand, the clinical Utility of VI-RADS has shown great promise in pre-TURBT assessment, providing a reliable method for predicting muscle invasion and guiding treatment decisions. As Del Giudice et al. pointed out, VI-RADS can potentially reduce the need for repeat TURBT, thereby minimizing patient morbidity and improving care pathways [11]. Additionally, this meta-analysis reaffirms the findings of studies by Liu et al. and Arita et al., which reported that the preoperative use of VI-RADS led to better treatment planning and outcomes in bladder cancer patients [23,26,39].

The use of a standardized scoring system such as VI-RADS not only facilitates better communication among multidisciplinary teams but also enhances the reproducibility of imaging results. For instance, Akcay et al. demonstrated that using standardized VI-RADS scoring improved inter-radiologist agreement, enabling more consistent application of the system across institutions [39]. This is crucial for ensuring that the VI-RADS system is widely adopted in clinical practice [11].

For cutoff values and diagnostic performance, while a VI-RADS cutoff score of ≥ 3 provided the highest diagnostic accuracy, some studies argued for using a cutoff of ≥ 4 to enhance specificity at the expense of sensitivity [11,39]). Del Giudice et al. found that a cutoff of ≥ 4 resulted in a specificity of 94%, compared to 84% for a cutoff of ≥ 3, making it particularly useful in settings where reducing false positives is critical [11]. However, Luo et al. observed that a higher cutoff might miss early cases of MIBC, underscoring the need to balance sensitivity and specificity based on clinical context [11,39,41].

## Clinical implications & limitations

The strong diagnostic performance of VI-RADS at a cutoff score of ≥ 3 supports its integration into routine clinical practice for the pre-TURBT assessment of bladder cancer. By using VI-RADS, clinicians can more accurately stage bladder cancer, reducing the need for repeat TURBT and minimizing patient morbidity [39]. This scoring system has the potential to expedite treatment decisions and improve outcomes for patients with MIBC, as also suggested by [11,22,26,39].

Despite the promising findings, several limitations were identified. First, most studies included in the meta-analysis were retrospective and single-centered, which could introduce selection bias. Retrospective designs often lack control over confounding factors, limiting the generalizability of the results. Second, there was moderate heterogeneity in specificity across studies, which may be due to differences in MRI protocols, radiologist expertise, and reference standards. The presence of publication bias, as detected by Deeks’ funnel plot, further complicates the interpretation of results. Lastly, while VI-RADS is effective for staging MIBC, it does not account for other important prognostic factors such as carcinoma in situ or histological variants. Nevertheless, as none of the studies evaluated the used VI-RADS score of 4 as a result we were not able to evaluate if the utilization of higher score improves sensitivity and specificity.

## Conclusion

In conclusion, the VI-RADS system demonstrates excellent diagnostic performance, especially with a cut-off score of ≥ 3, in detecting muscle-invasive bladder cancer. Its application in clinical practice has the potential to reduce the need for TURBT, minimize complications, and expedite treatment. Given the growing evidence supporting its use, VI-RADS should be considered for inclusion in standard bladder cancer staging guidelines. Further multi-center, prospective research is required to optimize its use across different radiological and clinical environments.
